# Medicinal herbs *Fructus corni* and *Semen cuscutae* suppress allograft rejection *via* distinct immune mechanisms

**DOI:** 10.18632/oncotarget.9680

**Published:** 2016-05-31

**Authors:** Xusheng Liu, Yu-Qun Zeng, Yong-Zhuo Liang, Chuan Zou, Huazhen Liu, Feifei Qiu, Chun-Lin Liang, Xiao-Wei Jin, Zi-Ren Su, Zhenhua Dai

**Affiliations:** ^1^ Department of Nephrology, the Second Clinical College, Guangzhou University of Chinese Medicine, Guangzhou, Guangdong, P.R. China; ^2^ School of Chinese Materia Medica, Guangzhou University of Chinese Medicine, Guangzhou, Guangdong, P.R. China; ^3^ Section of Immunology, Guangdong Provincial Academy of Chinese Medical Sciences, Guangzhou, Guangdong, P.R. China

**Keywords:** T cells, immunosuppression, herbs and transplantation, Immunology and Microbiology Section, Immune response, Immunity

## Abstract

Achieving long-term allograft survival without continuous global immunosuppression is highly desirable because constant immunosuppression causes severe side effects. Traditional Chinese medicine (TCM) has been utilized to treat numerous diseases for centuries. To seek novel immunosuppressive agents, we investigated several Chinese herbal formulas that have been shown to be effective in treating autoimmune diseases. C57BL/6 mice were transplanted with a skin graft from Balb/C donors and treated orally with the TCM. IL-12-expressing dendritic cells and CD4+FoxP3+ Tregs were quantified by flow cytometer while intragraft IL-12 gene expression was measured by real-time PCR. Here we identified a unique TCM, San Si formula, which contains three herbs: *Fructus corni* (FC), *Fructus ligustri lucidi* (FLL) and *Semen cuscutae* (SC). We found that either SC or FC, but not FLL, significantly prolonged skin allograft survival while SC plus FC or San Si formula further delayed allograft rejection compared to SC or FC alone. SC and FC, which did not contain cyclosporine and rapamycin, reduced graft-infiltrating T cells and suppressed their proliferation. Importantly, it was SC, but not FC, that induced CD4+FoxP3+ Tregs in recipients. Tregs induced by SC were also more potent in suppression. In contrast, FC repressed both intracellular IL-12 expression by intragraft DCs and IFNγ expression by graft-infiltrating T cells. Moreover, FC inhibited intragraft IL-12 gene expression. Depleting Tregs and providing exogenous IL-12 completely reversed allograft survival induced by SC plus FC. Thus, SC and FC synergistically suppress allograft rejection via distinct mechanisms.

## INTRODUCTION

An allograft is always rejected in the absence of continuously immunosuppressive treatments, which generally cause various side effects. Some conventional immunosuppressants also inhibit regulatory T cell (Treg) development and function, and therefore, hinder transplant tolerance. For instance, cyclosporine blocks IL-2 expression [1–3] and consequently compromises Treg survival and function [4–6]. Hence, achieving long-term allograft survival without long-term treatments with any global immunosuppressive agent is highly desired in organ transplantation.

Traditional Chinese medicine (TCM) has been utilized to effectively treat various diseases for centuries and has recently become a hot topic since Youyou Tu won a Nobel Prize due to her discovery of artemisinin, which was extracted from malaria-fighting Chinese herbal medicine *Weet wormwood*. Previous studies have also shown that TCM or its extracts can inhibit or prevent allergy, asthma, and rheumatoid arthritis in both humans and animal models [7–9]. Moreover, TCM can suppress alloimmune responses and allograft rejection [10–13]. Therefore, TCM could be a better option to prevent allograft rejection. However, extensive studies are needed to seek more effective TCM to inhibit allograft rejection. In particular, more in-depth investigations should be focused on the mechanisms underlying immunoregulation mediated by TCM. We investigated several Chinese herbal formulas that have never been published despite their clinical utilization. They have been shown to be highly effective in treating some autoimmune diseases according to testimonies of numerous patients as well as some of the well-recognized physicians specialized in TCM. Here we found that two herbal components from San Si formula, *Fructus corni* (FC) and *Semen cuscutae* (SC), significantly delayed murine skin allograft rejection. Treatments with a combination of both herbs further extended the allograft survival, whereas *Fructus ligustri lucidi* (FLL), another ingredient of San Si formula, did not significantly alter allograft survival. UFLC analyses demonstrated that San Si formula did not contain traditional immunosuppressive agents such as cyclosporine and rapamycin. It was FC that inhibited Th1 responses by repressing IL-12 expression in dendritic cells (DCs), whereas SC induced CD4+FoxP3+ regulatory T cells (Treg) that were more potent in suppression.

## RESULTS

### Treatments with SC and FC, but not FLL, significantly prolong skin allograft survival

**Figure 1:**
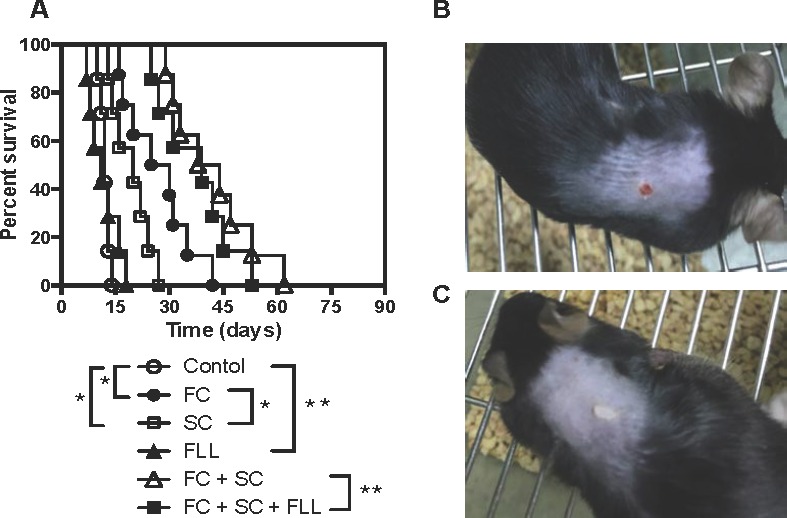
FC and SC prolong skin allograft survival Skin allografts derived from Balb/C mice were transplanted to C57BL/6 mice that were then treated with San Si formula or its components for four weeks as described in the method. Allograft rejection was then observed with seven to eight transplants per group **A.**. Shown also were a representative of rejected skin allografts in control recipients (Figure 1B) and one of accepted skin allografts in recipients treated with FC plus SC two weeks post-transplantation (Figure 1C). (*P < 0.05 and **p > 0.05, *n* = 7-8 mice).

Based on unpublished clinical effects of San Si formula on autoimmune diseases, we asked whether any of its components or itself also suppresses allograft rejection. Skin allografts derived from Balb/C mice were transplanted to C57BL/6 mice that were then treated with San Si formula or its components. As shown in Figure 1A, we found that either FC alone or SC alone significantly prolonged skin allograft survival compared to the control (FC: MST = 28 *vs*. 12 days and SC: MST = 20 *vs*. 12 days) while FC plus SC further extended allograft survival (MST = 41 *vs*. 12 days). In contrast, FLL alone did not delay skin allograft rejection compared to the control (MST = 11 *vs*. 12 days). Moreover, FLL plus both FC and SC also did not further extend allograft survival compared with FC plus SC (MST = 39 *vs*. 41 days), although treatments with FLL plus both FC and SC, all of the San Si components, prolonged allograft survival. We chose the dose of 6.5 g/kg according to its clinical usage that did not result in side effects in patients, and our preliminary studies demonstrated that this dosage did not cause any weight loss, illness and toxic injuries to a murine kidney and liver, as proofed by laboratory tests of renal and liver functions (data not shown). Shown also were a representative of a rejected skin allograft in control recipients (Figure 1B) and one of accepted skin allografts in recipients treated with FC + SC (Figure 1C).

### SC and FC suppress graft-infiltrating T cell proliferation *in vivo*

**Figure 2:**
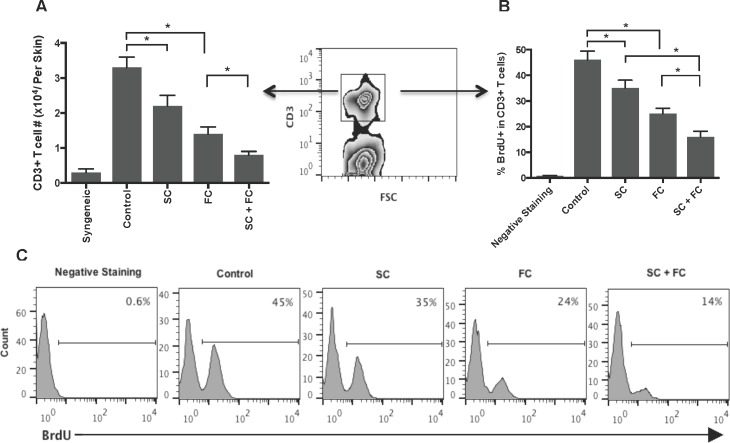
SC and FC suppress graft-infiltrating T cell proliferation and expansion B6 mice were transplanted with a Balb/C skin graft and treated with San Si components. One week later, graft-infiltrating cells were isolated and CD3+ T cells were enumerated by FACS analyses **A.**. CD3+ T cell proliferation was also measured by BrdU uptakes **B.** & **C.**. Data for A and B are presented as Mean ± SD. Histograms are gated on CD3+ cell populations. One of three separate experiments is shown. (*P < 0.05, *n* = 4-5 mice/group).

To determine whether San Si formula inhibits alloreactive CD3+ T cell proliferation *in vivo*, transplanted mice were treated with SC and/or FC. One week later, graft-infiltrating cells were isolated, enumerated for CD3+ T cell components by FACS, and analyzed for T cell proliferation by BrdU uptakes. As shown in Figure 2A, either SC alone or FC alone significantly reduced the numbers of CD3+ T cells infiltrating skin allografts while SC plus FC further lowered their numbers. Similarly, either SC alone or FC alone significantly decreased the percentage of BrdU+ T cells while SC plus FC largely abolished BrdU+ T cells compared to the control group (Figure 2B and 2C). However, FC was more effective in the suppression of T cell proliferation or reduction in T cell numbers than SC. These findings suggest that two herbs, SC and FC, can synergistically suppress T cell proliferation *in vivo*.

### SC and FC do not contain conventional immunosuppressants cyclosporine and rapamycin

**Figure 3:**
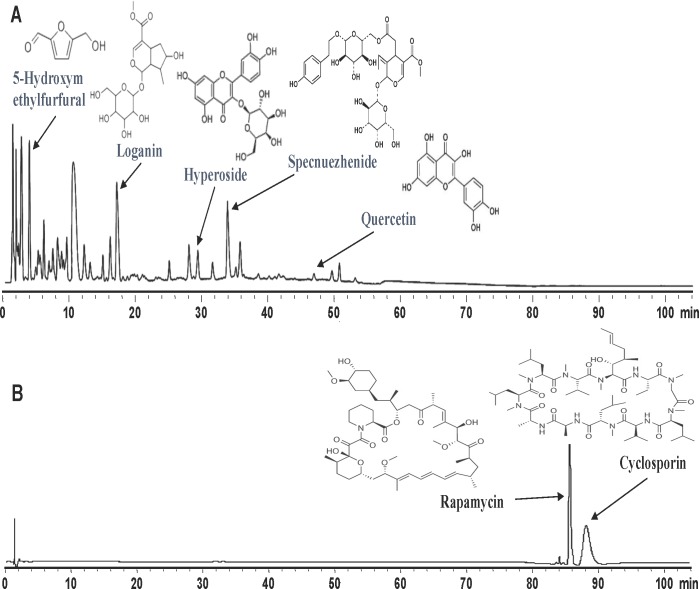
Generation of UFLC (Ultra Fast Liquid Chromatography) fingerprints San Si formula and all standard samples **A.**, including Loganin, Hyperoside, Specnuezhenide, Quercetin and 5-Hydroxymethylfurfural, as well as immunosuppressive agents Cyclosporin and Rapamycin **B.** were subject to UFLC analyses using a Shimadzu UFLC system consisting of LC-20AD pump, photodiode-array detector and LC solution chromatographic workstation. One of two independent experiments was shown. The results suggest that the entire San Si formula does not contain traditional immunosuppressive agents cyclosporine and rapamycin.

Since SC and FC inhibit alloimmune responses and allograft rejection, it is imperative to rule out that they contain an ingredient of conventional immunosuppressants such as cyclosporine and rapamycin, which otherwise would be responsible for the suppression of allograft rejection by SC and/or FC. To this end, we generated UFLC fingerprints of San Si formula with control samples, including both cyclosporine and rapamycin. As shown in Figure 3, peaks for San Si were overwhelmingly located within 50 min while the peaks for cyclosporine and rapamycin fell in the range of 85 to 90 min, suggesting that San Si formula, including SC, FC and FLL, do not contain any ingredient of cyclosporine or rapamycin.

### SC, but not FC, induces CD4+FoxP3+ Tregs

**Figure 4:**
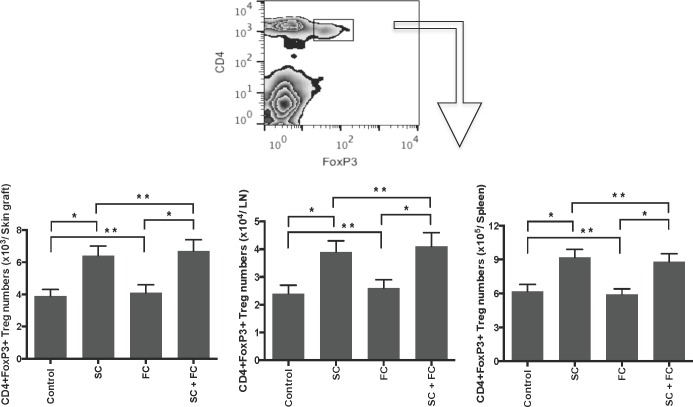
SC, but not FC, induces CD4+FoxP3+ Tregs B6 mice were transplanted with a Balb/C skin graft and treated with SC and/or FC. Draining LN, spleen and graft-infiltrating cells were isolated and CD4+FoxP3+ Tregs were enumerated by FACS analyses one week following transplantation. Data are presented as Mean ± SD. One representative of three separate experiments is shown. (*P < 0.05 and **p > 0.05, *n* = 4-5 mice/group).

Since CD4+FoxP3+ Tregs are essential for suppression of alloimmune responses, we asked whether these herbs also inhibit allograft rejection by inducing Tregs. Draining LN, spleen and graft-infiltrating cells were isolated and CD4+FoxP3+ Tregs were enumerated by FACS analyses one week following transplantation. As shown in Figure 4, SC significantly augmented the number of CD4+FoxP3+ Tregs in skin allografts, draining LNs and spleens while FC did not. SC increased CD4+FoxP3+ Tregs in all locations, suggesting that it generally expands Tregs. Moreover, SC plus FC did not further increase Treg numbers when compared to SC alone, confirming that FC does not promote Treg generation. Similar changes of Treg numbers with SC and FC treatments occurred two weeks following transplantation (data not shown).

**Figure 5:**
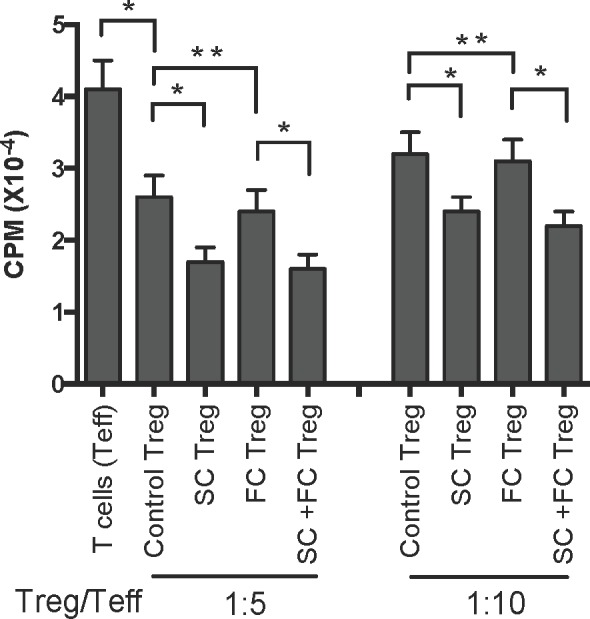
*In vitro* suppressive capacity of SC-induced Tregs derived from recipient mice One-way MLRs were set up using T cells (Teff) from B6 mice as responders and irradiated donor-derived Balb/C splenocytes as stimulators with or without CD4+CD25+ Tregs derived from transplanted B6 mice that were treated with SC and/or FC. The ratio of Treg : Teff was 1:5 and 1:10 respectively. Data are presented as Mean ± SD. One representative of three independent experiments is shown. (*P < 0.05 and **p > 0.05, *n* = 3-5 mice/group).

To examine if SC also enhances Treg suppressive function, one-way MLRs were set up using T cells from naïve B6 mice as responders and irradiated donor Balb/C splenocytes as stimulators, together with CD4+CD25+ Tregs as regulators, which were isolated from transplanted B6 mice treated with SC and/or FC one week post-transplantation. As shown in Figure 5, at the ratio of 1:5 (Treg/Teff), Tregs from transplanted control mice without herb treatments (Control Treg) indeed suppressed T cell proliferation compared to T cells alone group (Teff) without Tregs. Moreover, Tregs from SC-treated recipients (SC Treg) further inhibited T cell proliferation compared to the control Tregs, whereas Tregs from FC-treated recipients (FC Treg) did not do so when compared to the control Tregs. Similar findings were also seen at the ratio of 1:10 (Figure 5), suggesting that CD4+FoxP3+ Tregs induced by treatments with SC are more potent in suppression than control Tregs from transplanted recipients.

### FC, but not SC, inhibits IL-12 expression by graft-infiltrating DCs as well as allografts

**Figure 6:**
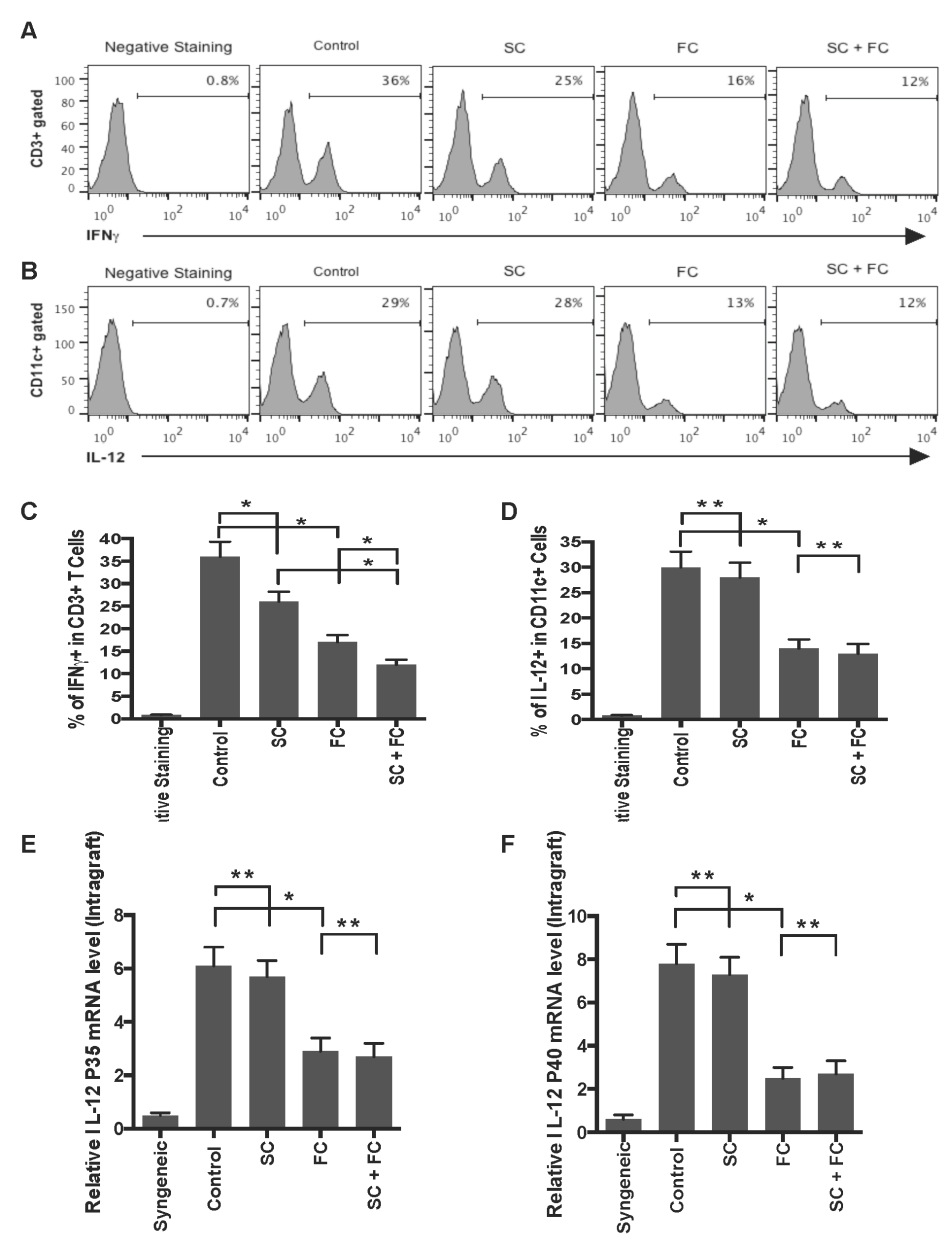
FC inhibits IL-12 expression by graft-infiltrating DCs as well as allografts The similar graft-infiltrating cells were isolated and stained with anti-IL-12 (P40/70) and anti-CD11c or anti-IFNγ and anti-CD3 Abs one week post-transplantation. The intracellular expressions of IFNγ within CD3+ population **A.** & **C.** and IL-12 within CD11c+ population **B.** & **D.** were measured by FACS analyses, with total RNA being isolated from skin allografts to measure gene expressions of IL-12 P35 **E.** and IL-12 P40 **F.**. Histograms were gated on either CD3+ **A.** or CD11c+ population **B.**. Data are presented as Mean SD. One representative of three independent experiments is shown. (*P < 0.05 and **p > 0.05, *n* = 4-5 mice/group).

Since SC and FC prolonged allograft survival in this study, they could suppress allograft rejection by dampening a Th1 response. To test this hypothesis, graft-infiltrating cells were isolated and stained with anti-IL-12 (P40/70) and anti-CD11c or anti-IFNγ and anti-CD3 Abs one week post-transplantation. Their intracellular expressions of IL-12 within CD11c+ population and IFNγ within CD3+ population were determined by FACS analyses, with total RNA being isolated from skin allografts to measure IL-12 gene expression. As shown in Figure 6A and 6C, either SC alone or FC alone significantly reduced the percentage of IFNγ+ graft-infiltrating T cells while SC plus FC further lowered their percentage, indicating that both SC and FC suppress Th1 cell effector function. In contrast, only FC, but not SC, decreased the percentage of IL-12+ graft-infiltrating CD11c+ DCs (Figure 6B and 6D). Moreover, SC plus FC did not further reduce their percentage when compared to FC alone. Therefore, it was FC that inhibited a Th1 response by repressing IL-12 expression in DCs. Similarly, FC, but not SC, suppressed intragraft gene expression of both IL-12P35 and IL-12P40 (Figure 6E and 6F) while SC plus FC did not further reduce IL-12 mRNA levels when compared to FC alone. Our findings demonstrated that FC, but not SC, represses IL-12 expression at both the gene and protein levels.

### Administration of rIL-12 or depletion of CD25+ Tregs shortens allograft survival induced by treatments with SC and FC

**Figure 7:**
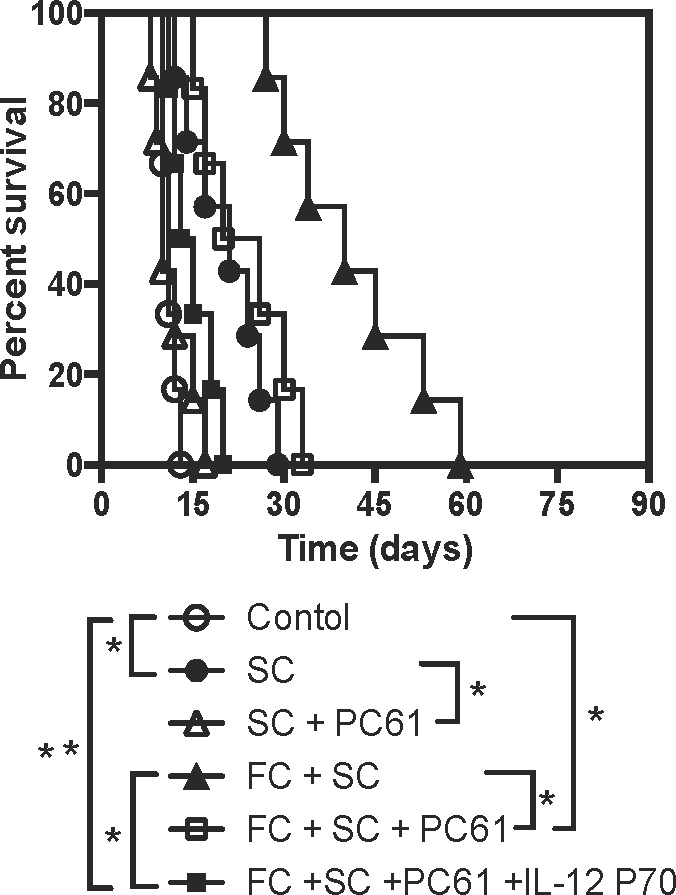
Depleting CD25+ Tregs and administering rIL-12 reverse skin allograft survival induced by SC and FC B6 mice were transplanted with a Balb/C skin graft and treated with SC and/or FC. Some recipient mice were also treated with CD25+ Treg-depleting Ab PC61 and/or recombinant rIL-12 P70. Depletion of the Tregs and administration of recombinant murine rIL-12 completely reversed skin allograft survival induced by SC plus FC. (*P < 0.05 and **p > 0.05, *n* = 6-7 mice).

Given that SC induced CD4+FoxP3+ Tregs and that FC suppressed IL-12 expression, we examined if depletion of Tregs and/or administration of recombinant rIL-12 would reverse the allograft survival induced by SC and/or FC. As shown in Figure 7, depletion of Tregs with PC61 abolished the extension of skin allograft survival induced by SC alone, and also significantly shortened skin allograft survival induced by SC plus FC while depleting Tregs together with administration of rIL-12 (P70) completely reversed the prolongation of allograft survival induced by SC plus FC. As controls, isotype Ab for PC61 did not alter skin allograft survival while administration of rIL-12 alone also did not accelerate skin allograft rejection (data not shown). Moreover, treatments with Treg-depleting Ab, PC61, achieved ~80% of Treg deletion *in vivo*, as described in our previous studies [14, 15].

## DISCUSSION

Achieving long-term allograft survival without continuous global immunosuppression is highly desired in organ transplantation because long-term global immunosuppression causes various adverse reactions. Traditional Chinese medicine (TCM) has been utilized to treat various diseases, including autoimmune diseases, for centuries and could provide a better option for suppressing allograft rejection. It has recently become a hot topic since Youyou Tu won a Nobel Prize due to her discovery of artemisinin, which was extracted from malaria-fighting Chinese herbal medicine *Weet wormwood*. TCM is often referred to as “evidence-based” or “medicinal treasury” given that it has been discovered and accumulated over hundreds of years and that patients can still rely on it even when many modern drugs are not working. It represents an untapped source of potentially effective medications and provides valuable clues to where to look for a new drug. What is compelling is to standardize its therapeutic protocols and unveil its exact mechanisms of action. In this study, we found that two medicinal herbs, SC and FC, significantly prolonged skin allograft survival. SC and FC, which did not contain any cyclosporine and rapamycin according to UFLC analyses, diminished graft-infiltrating T cells and suppressed their proliferation. Furthermore, it was SC, but not FC, that induced CD4+FoxP3+ Tregs. In contrast, FC, but not SC, repressed intracellular IL-12 expression by intragraft DCs. Thus, FC and SC synergistically suppress allograft rejection *via* different mechanisms. It remains unknown whether they also inhibit rejection of a vascularized allograft. We propose that they do since a skin transplant model is more stringent than a vascularized one.

Although TCM from “medicinal treasury” may provide vast opportunities for new drug discoveries, especially in terms of where to get started, much work needs to be done before more significant progresses can be made. In particular, more in-depth studies are required to reveal the exact mechanisms of its action. Among several “autoimmunity-suppressing” TCM formulas we screened, we only found that San Si itself or its components FC and SC suppressed allograft rejection. One of its ingredients, FLL, either alone or in combination with FC plus SC, did not further inhibit allograft rejection, implying that FLL is not a necessary component of San Si formula for its suppression of alloimmune responses and, perhaps, autoimmunity. In fact, FLL was previously reported to enhance proliferative activity of piglet blood lymphocytes and upregulate CD4+ T cell population [16]. Hence, it is unclear why FLL was included in San Si formula. However, previous studies have shown that FC indeed inhibits airway inflammation and hyper-responsiveness in a mouse model of allergic asthma [17] and that ursolic acid isolated from FC ameliorates murine colitis [18], indicating that FC is immunosuppressive or anti-inflammatory. While an ingredient of San Si formula, FLL, was not working in our transplant models, we also tried other “autoimmunity-suppressing” formulas used by local physicians specialized in TCM and found that those formulas did not suppress alloimmunity either (our unpublished observation). Our findings suggest that a significant discovery of a new drug derived from TCM still requires extensive screening. In particular, continuous efforts are warranted to isolate and characterize the active compounds or ingredients within San Si formula or its components FC and SC, although it could take a decade to finally accomplish this goal. Fortunately, we ruled out that San Si formula contains a popular global immunosuppressant such as cyclosporine or rapamycin. Thus, it's imperative to identify actual compounds within FC and SC that are actually responsible for suppression of allograft rejection. However, it is also possible that net effects of FC and SC require cooperation between many of the small molecules or compounds within these two herbs, instead of just a few of the molecules.

Interestingly, we found that FC and SC prolonged allograft survival through a different mechanism. FC suppressed Th1 responses *via* inhibition of IL-12 expression in DCs and IFNγ expression by graft-infiltrating T cells. It has been well known that allograft rejection is mainly mediated by Th1 responses [19, 20]. Therefore, FC suppresses allograft rejection *via* dampening Th1 responsiveness. On the other hand, we found that SC induced CD4+FoxP3+ Tregs in recipient mice. It has also been known that induction of endogenous Tregs or the adoptive transfer of exogenous Tregs prevents autoimmunity and allograft rejection in animal models [21–28]. Hence, SC inhibits allograft rejection *via* promoting Treg generation. In addition, SC also enhanced Treg suppressive function since SC-induced Tregs were more potent in suppression than control Tregs. It remains to be defined why SC-induced Tregs are more effective in suppression. It is likely that SC treatments generate more Ag-specific Tregs. It is also unclear whether other mechanisms are involved in the suppression of allograft rejection by FC and SC.

In conclusion, we have demonstrated that two popular Chinese medicinal herbs, FC and SC derived from original San Si formula, suppress allograft rejection in mice in the absence of any additional immunosuppressive treatment. FC and SC inhibit alloimmune responses *via* a distinct mechanism and, hence, synergistically exert their suppressive functions. These findings provide new insight into the immunosuppressive and anti-inflammatory effects of TCM and necessitate the continued characterization and testing of these two herbs. Our results will also lay the groundwork for clinical trials using TCM in transplanted patients, especially those who have developed severe side effects resulted from long-term immunosuppressive treatments. More significant discoveries of TCM are expected due to ongoing efforts to study TCM in China.

## MATERIALS AND METHODS

### Ethics statement

Investigation has been conducted in accordance with the ethical standards and according to the Declaration of Helsinki and according to national and international guidelines and has been approved by the authors’ institutional review board.

### Mice and reagents

Wild-type BALB/c and C57BL/6 mice were purchased from Guangdong Medical Laboratory Animal Center (Fushan, Guangdong, China). All mice were housed in a specific pathogen-free environment and all animal experiments were approved by the Institutional Animal Care and Use Committee of Guangdong Provincial Academy of Chinese Medical Sciences. Anti-FoxP3-PE mAb was bought from eBioscience (San Diego, CA) while recombinant murine IL-12P70, anti-CD4-FITC, anti-CD11c-PE, anti-CD3-PE, anti-CD3-FITC, anti-IL-12-APC (p40/p70), anti-BrdU-FITC, anti-IFNγ-PE, and purified, depleting anti-CD25 (PC61) mAbs were purchased from BD Biosciences (Bedford, MA).

### Preparation of herbal decoction

Chinese herbs San Si formula, including *Fructus corni* (FC), *Semen cuscutae* (SC) and *Fructus ligustri lucidi* (FLL), were purchased from Kangmei Pharmaceutical Co. Ltd. (Punin, Guangdong, China). All herbal decoctions were prepared according to standard procedures [29], and all of the procedures were in accordance with the demand in 2010 edition of China Pharmacopoeia. Water extracts were then concentrated and dried out with a rotary evaporator under vacuum.

### Generation of UFLC (ultra fast liquid chromatography) fingerprints

Standard samples Loganin, Hyperoside, Specnuezhenide, Quercetin, 5-Hydroxymethylfurfural, Cyclosporin, and Rapamycin were purchased from National Institutes for Food and Drug Control (Beijing, China). Acetonitrile (LC grade) was bought from Merck (Darmstadt, Germany). All standard samples and San Si compounds were dissolved with methanol and filtered through a 0.45μm filter membrane before analyses. A Shimadzu UFLC system (Kyoto, Japan) consisting of LC-20AD pump, photodiode-array detector and LC solution chromatographic workstation was used for the analyses. The chromatographic separation was carried out on Synergi Hydro-RP (150 mm×2.00 mm, 4μm, Phenomenex Inc., USA). The flow rate was 0.4ml/min and sample injection volume was 10μl. Detection wavelength was set at 254 nm with a column temperature at 38°C. Mobile phase contained solvent A (acetonitrile) and B (water/methanoic acid, 100:0.1, v/v). The gradient elution was optimized as followings: a linear gradient of 5-10% A (0-12 min), a linear gradient of 10-25% A (12-50 min), a linear gradient of 25-32% A (50-70 min), a linear gradient of 32-80% A (70-85 min), a linear gradient of 80-5% A (85-100 min), and a linear gradient of 5-5% A (100-105 min). The system was then restored to initial conditions. UFLC fingerprints of Shan Si formula and the standards were shown in Figure 3.

### Skin transplantation

Skin donors were 7-8-week-old wild-type BALB/c mice, and skin allograft recipients were 7-8-week-old C57BL/6 mice. Full-thickness trunk skin was transplanted to the dorsal flank area of recipient mice and secured with the bondage of Band-Aid (Johnson Johnson, New Brunswick, NJ). Skin rejection was defined as graft necrosis greater than 90% as described in our previous publications [15, 30].

### Isolation of graft-infiltrating cells

Skin allografts were removed, minced and digested at 37°C for 30 min in 20ml RPMI-1640 medium containing 10% FCS and 250u/ml collagenase (Sigma, St. Louis, MO). Cells were washed twice with HBSS after digestion. To clear the debris, cell suspensions were rapidly passed down a loosely packed glass wool column (300 mg sterile glass wool in a 10ml syringe) and a hand-held 70 μm nylon cell strainer (BD Biosciences), then mixed with Percoll solution (Sigma) to a concentration of 30%, and centrifuged at 2000 rpm for 20 minutes at room temperature. The pellet was washed and re-suspended before FACS analyses.

### Treatments of mice

San Si formula, including SC, FC and FLL, or its component was dissolved in distilled water and orally administered at 6.5 g/kg/mouse daily for four weeks post-transplantation or until allograft rejection/collection. The herbal dosage was calculated based on the clinical usage that did not cause side effects in patients. In some recipients, recombinant murine rIL-12 (P70) was administered at 2 μg/day on days 2, 4, 6, 8, 10 and 14, while depleting anti-CD25 Ab (PC61) was used at 0.1 mg on days 0, 2, 4, 6, 8 and 10 following transplantation, as described in our previous studies [14, 15].

### Analyses of T cell proliferation *in vivo* by 5-Bromo-2′-Deoxyuridine (BrdU) labeling

Recipient mice were pulsed *i.p*. with 0.8 mg of BrdU (Sigma, St. Louis, MO). 24 hours later, skin graft-infiltrating cells were isolated and first stained with anti-CD3-PE Ab. Cells were then fixed in 70% ethanol, followed by 2% paraformaldehyde, and incubated with 50 Units/ml of DNase I (Sigma). Cells were finally stained with anti-BrdU-FITC and analyzed by a FACSCalibur (BD Biosciences), as described previously [15, 31].

### Analyses of T cell proliferation *in vitro* for Treg suppression assays

CD4+CD25+ Tregs were sorted out from transplanted B6 mice that were treated with herbs SC, FC or SC plus FC. They were then cultured with nylonwood-enriched T cells (Teff), isolated from naïve B6 mice, in 96-well plates in the complete RPMI 1640 medium (10%FCS, 2mM glutamine, 100U/ml penicillin, and 100μg/ml streptomycin). The ratios of Treg:Teff are 1:5 and 1:10, respectively. Irradiated BALB/c spleen cells were added to the culture to serve as donor-derived stimulators, as described previously [15, 31]. Four days later, cells were harvested and analyzed by a Scintillation counter (PerkinElmer, Meriden, CT). Cells were pulsed with [^3^H]-Thymidine for the last 8 hours.

### Purification of CD4+CD25+ T cells by FACS cell sorting

B6 mice were transplanted with a Balb/C skin graft and treated with the TCM as described above. Spleen cells from recipient mice were pooled after lysing red blood cells. Cells were then stained with anti-CD4-FITC and anti-CD25-PE mAbs, and CD4+CD25+ Tregs were sorted out using FACSAria III (BD Biosciences). The purity of sorted cells was typically > 95%.

### Intracellular staining and flow analyses

Draining lymph node, spleen and graft-infiltrating cells were isolated as described previously [32, 33]. Cells were stained first for surface markers with anti-CD4-FITC, anti-CD11c-PE or anti-CD3-FITC, and then intracellular markers with anti-FoxP3-PE, anti-IL-12 (P40/P70)-APC or anti-IFNγ-PE using intracellular staining fixation & permeabilization kit (eBioscience). CD4+FoxP3+Tregs, CD11c+IL-12+ or CD3+IFNγ+ cells were enumerated by FACS analyses.

### Measurement of gene expressions by Real-time PCR

Skin allografts were harvested, and total RNA was extracted with the RNeasy kit (Qiagen). The reverse transcription was performed using Multiscribe Reverse Transcriptase Enzyme (Applied Biosystems, Foster City, CA). Real-time PCR was performed using an ABI 7700 sequence detector system (Applied Biosystems). To measure mRNA levels, we normalized the expression of target genes to the housekeeping gene HPRT, and data were represented as relative expression of the target gene to the housekeeping gene. The primers were used as follows: IL-12p35 sense, 5-CACGCTACCTCCTCTTTTTG-3; IL-12p35 anti-sense, 5-CAGCAGTGCAGGAATAATGTT-3; IL-12p40 sense, 5-AAACCAGACCCGCCCAAGAAC-3; IL-12p40 anti-sense, 5-AAAAAGCCAACCAAGCAGAAGACAG-3; HPRT sense, 5-GGTTAAGCAGTACAGCCCCAAAAT-3; and HPRT anti-sense, 5 -ATAGGCACATAGTGCAAATCAAAAGTC-3.

### Statistical analysis

Comparisons of the mean were performed using ANOVA. The analysis of graft survival was conducted using Kaplan-Meier method (log-rank test). All analyses were performed using Prism-6 software (GraphPad Software, La Jolla, CA). Data were presented as Mean ± SD. A value of *P* < 0.05 was considered statistically significant.

## Abbreviations

DC: dendritic cell
FC: fructus corni
FLL: fructus ligustri lucidi
MST: median survival time
SC: semen cuscutae
TCM: traditional Chinese medicine
Treg: regulatory T cell
and UFLC: Ultra Fast Liquid Chromatography.
